# Predictors of Changes in Quality of Life of Patients with Major Depressive Disorder—A Prospective Naturalistic 3-Month Follow-Up Study

**DOI:** 10.3390/jcm12144628

**Published:** 2023-07-12

**Authors:** Vlad Dionisie, Maria Gabriela Puiu, Mirela Manea, Ioana Anca Pacearcă

**Affiliations:** 1 Department of Psychiatry and Psychology, “Carol Davila” University of Medicine and Pharmacy, 020021 Bucharest, Romania; vlad.dionisie@gmail.com (V.D.); mirelamanea2003@yahoo.com (M.M.); 2 “Prof. Dr. Alexandru Obregia” Clinical Hospital of Psychiatry, 041914 Bucharest, Romania; 3 Doctoral School, “Carol Davila” University of Medicine and Pharmacy, 020021 Bucharest, Romania; ioanapacearca@gmail.com; 4 “Sfântul Spiridon Vechi” Foundation, 040012 Bucharest, Romania

**Keywords:** major depressive disorder, quality of life, treatment intervention, functionality, pain, WHOQOL-BREF, visual analogue scale, Sheehan Disability Scale, patient reported outcomes, follow-up

## Abstract

Major depressive disorder (MDD) is one of the leading causes of disease burden worldwide and affected patients frequently report impairments in quality of life (QoL). Therefore, the present research aimed to identify predictors of domain-specific QoL changes in MDD patients following the acute phase of pharmacological treatment (3-month). This study is a prospective, naturalistic, and observational analysis on 150 patients. Depressive symptoms, QoL, overall pain intensity, and functionality were assessed using Hamilton Depression Rating Scale, World Health Organization Quality of Life scale—abbreviated version, Visual Analog Scale, and Sheehan Disability Scale, respectively. Reductions in symptom severity and disability were predictors of improvement across all domains of QoL. Pain intensity reduction was a predictor of increases in the physical aspect of QoL. A reduced number of psychiatric hospitalizations and being in a relationship predicted an improvement of QoL in the psychological domain whereas a positive history of suicidal attempts was associated with better social relationships QoL. The predictive models explained 41.2% and 54.7% of the variance in psychological and physical health domains of QoL, respectively. Awareness of sociodemographic and changes in clinical factors that impact the change in domain-specific QoL might help in shaping personalized treatment.

## 1. Introduction

Depressive disorders are a major public health concern and a leading cause of non-fatal disease burden worldwide as measured by years lived with disability [1]. Major depressive disorder (MDD) is characterized by different mood, cognitive, and behavioral symptoms leading to several functional impairments [2,3]. Consequently, quality of life (QoL) is seriously affected in patients with MDD, frequently even more than in those with medical conditions, as shown by numerous studies [4,5,6,7]. For several decades, research has concentrated on symptoms remission as the main goal of different pharmacological or psychological treatments in depression. In the last decade, quality of life has emerged as an important outcome in MDD treatments [8,9]. In addition, the patient perspective revealed that some outcomes beyond symptoms recovery, such as QoL, returning to the pre-morbid level of functioning, and lack of depression-related pain, would be more important [10,11,12,13]. QoL is defined by the World Health Organization (WHO) as “an individual’s perception of his or her position in life in the context of the culture and value systems in which he or she lives, and in relation to his or her goals, expectations, standards, and concerns” [14]. Therefore, from this standpoint, QoL is a broad and complex concept and refers to an individual’s perception of multiple aspects of his or her own life, such as well-being, social relationships, satisfaction with life, physical and psychological health, living standards, and functionality [15,16].

The severity of depressive symptomatology was associated with greater impairments in QoL [7,17], but changes in symptoms intensity did not fully account for QoL improvements over time [7,18,19]. Moreover, while QoL improved substantially during the acute phase of treatment of MDD, it remained lower during the remission phase in comparison with healthy individuals [4,20,21]. In another study, QoL was significantly ameliorated independently of depression severity at week 4 [22]. Moreover, a large proportion of patients considered remitted from the clinician perspective did not consider themselves to be in remission and continued to have functional disabilities and impaired QoL [23]. This is an important pitfall of MDD treatment trials, and other variables, such as functioning and quality of life, should be included in the outcome assessment in MDD [24].

A recent meta-analysis of double-blind, placebo-controlled, randomized controlled trials revealed a moderate effect on QoL of antidepressant treatment [25]. The analysis of the Medical Expenditures Panel Survey from the United States [26] reported opposite results but this study was much criticized, and methodological and data interpretation flaws have been outlined [27]. Andrade C. (2022) emphasized once more that antidepressants improve QoL during the acute phase of treatment and suggested that impairments in recovered patients might be due to the context of their own life that cannot be treated by antidepressants [27].

Studies have found that QoL changes are associated with different sociodemographic and clinical factors as well. A greater income, absence of suicide thoughts, satisfaction with medication, history of prior antidepressant treatment, and older age at depression onset were associated with a positive change in QoL, while an increased number of adverse effects or of previous medication trials, increased disability, relapse of MDD, and living alone were associated with a worse QoL changes over time [22,28,29,30,31,32].

There is a large volume of published studies describing the complex relationship between unexplained painful physical symptoms and MDD [33]. Besides their frequent co-occurring, pain and depression are intertwined by multiple shared pathophysiological molecular mechanisms [34]. Higher pain intensity was correlated with more severe depressive symptoms and lower QoL [35]. In addition, Novik et al. (2013) observed that the presence of painful symptoms predicted a worse QoL change at 3-month follow-up compared with patients that did not have pain [36]. Moreover, the severity of pain at baseline and the degree of alleviation of pain were reported as predictors of changes in several QoL domains [37]. MDD is also commonly associated with chronic pain conditions and pain had a mediating effect between chronic conditions and the development of depression [38]. Vice versa, symptoms of depression determined an increased risk of developing chronic pain [39]. Moreover, suicidality is higher in individuals with chronic pain [40]. With respect to QoL, it was shown that regardless of the presence of MDD, patients suffering from chronic pain had lower overall QoL and that depression further decreased QoL [41,42].

Functional recovery is another important outcome closely linked to QoL as it appeared to mediate the relationship between depressive symptoms and QoL [43]. Moreover, Sheehan et al. (2017) reported in a systematic review that even though remission of depression was correlated with improvements in functionality, patients still experienced a certain level of disability [44]. Therefore, functional outcomes are recommended to be included in real-world clinical care and research designs [45].

The traditional model for the typical course of a major depressive episode consists of three illness stages (i.e., acute, remission, recovery). Correspondingly, the treatment phases can be divided as follows: acute, continuation, and maintenance. According to current guidelines, which support a 2-phase treatment model (i.e., acute and maintenance phases), the acute phase duration of MDD treatment is between 8 to 12 weeks. Successful acute treatment phase is essential for remission, which consists of patient being asymptomatic and with a certain degree of improvement in functionality [46,47].

Therefore, taking into account the maze of interactions between different factors in determining the QoL of patients with MDD, the present study was designed to identify the sociodemographic, clinical, functional, and pain-related predictors of change in the QoL of patients with MDD during the acute phase of treatment (i.e., 3 months) in a real-world setting.

## 2. Materials and Methods

### 2.1. Study Design and Population

This study had a prospective, naturalistic, and observational design and was conducted on a cohort of 250 adult patients with a DSM-5 [48] diagnosis of MDD. Participants were recruited from the inpatient and outpatient units of “Prof. Dr. Alexandru Obregia” Clinical Hospital of Psychiatry in Bucharest, Romania. The research was carried out between 1 July 2017 and 4 June 2019. The study was approved by the local Institutional Ethics Committee (approval no. 4224/23 February 2015) and followed the ethical principles of the Declaration of Helsinki for Medical Research involving human subjects. All patients provided written informed consent.

The present research had two time points. Baseline assessment (time point 0—T0) and follow-up (time point 1—T1) occurred at the moment of recruitment and 3 months after, respectively. Baseline assessment took place during the first three days of inpatient admission or during outpatient visit/consultation. At baseline, patients provided sociodemographic, personal, and clinical data. In addition, at baseline (T0), as well as 3 months after (T1), patients completed questionnaires (self or clinician rated) assessing depressive symptoms, quality of life, pain, and disability. All participants were evaluated individually and in-person at both study time points. Participants who were not present for assessment at T1 were considered lost to follow-up. All assessments in this study were carried out in the Romanian language.

Patients were included in the current study if they met the inclusion and exclusion criteria. We applied the following inclusion criteria: (1) age between 18–65 years old; (2) being admitted to one of the adult inpatient units or attending one of the outpatient units of “Prof. Dr. Alexandru Obregia” Clinical Hospital of Psychiatry; (3) meeting the DSM-5 criteria for MDD; (4) the patient did not received any psychopharmacological treatment for a minimum period of 3 months or the patient had taken antidepressant psychopharmacological treatment on which no changes have been made for at least 3 months (i.e., the treatment remained the same for at least 3 months); (5) the patient agreed to participate in the study and signed the informed consent; and (6) starting antidepressant pharmacological treatment or changing the previous antidepressant treatment regimen at the moment of recruitment. The exclusion criteria consisted of: (1) presence of psychotic symptoms; (2) the occurrence of depressive disorder is attributable to a medical condition; (3) receiving psychological treatment (psychotherapy); (4) serious medical illness (e.g., any type of cancer, cardiac, renal, or hepatic failure, cerebrovascular disease, autoimmune disease); (5) use of illicit drugs; (6) pregnant or lactating women; (7) the patient did not consent to participate and/or did not sign the informed consent; (8) patients with history of other current psychiatric comorbidities, including active alcohol or other psychoactive substances dependence (e.g., bipolar disorder, schizophrenia, alcohol used disorder, neurocognitive disorder, eating disorder, obsessive-compulsive disorder, etc.); and (9) patients involuntarily admitted.

The diagnosis of MDD according to DSM-5 criteria was determined after a comprehensive psychiatric evaluation made by a specialist licensed psychiatrist. The psychiatric comorbidities were excluded by the investigators during the psychiatric evaluation, based on the psychiatric history provided by the patients and based on each patient’s paper-based and electronic file from the hospital database.

The psychopharmacological intervention was determined solely by the attending psychiatrist of each patient and the authors of this study did not interfere in any way with the patient’s treatment regimen. The psychopharmacological intervention consisted of initiation of treatment in drug-naïve patients or of changes made to the treatment regimen the patient was already taking (e.g., increasing dose, switching to a different drug). In brief, patients received monotherapy with antidepressant medication or different combinations of antidepressants with mood stabilizers, anxiolytics, or antipsychotics. This study sought to determine the predictors of pharmacological treatment outcome (i.e., changes in WHOQOL-BREF domains) in a real-world setting and not the effect of a particular intervention.

### 2.2. Measures

#### 2.2.1. Sociodemographic, Personal, and Clinical Variables

Sociodemographic, personal, and clinical data were recorded at baseline for each patient using a semi-structured questionnaire especially designed for this research. The sociodemographic and clinical data retrieved included age (years), gender (female or male), educational level (≤8, 9–12 or >12 years), marital status (with or without partner), psychiatric family history (yes or no), age at first episode (years), illness duration (years), number of psychiatric hospitalizations, history of suicide attempts (yes or no), height (cm), weight (kg), receiving psychopharmacological treatment at the time of enrolment (yes or no), professional status (active or inactive professionally), income (<national minimum income, between national minimum and average income, ≥national average income), residence location type (rural or urban), and history of other psychiatric disorders (yes or no).

#### 2.2.2. Instruments

All participants were administered a set of psychometric instruments at baseline (T0) and 3 months after (T1) as well. The Romanian versions of the following psychometric scales were used: Hamilton Depression Rating Scale (HAM-D), World Health Organization Quality of Life scale—abbreviated version (WHOQOL-BREF), Visual Analog Scale (VAS) for pain, and Sheehan Disability Scale (SDS).

HAM-D is one of the most worldwide used clinician-administered scales to quantify the severity of depressive symptoms [49]. This scale was developed to evaluate the severity of depressive symptoms and it does not serve as a diagnostic instrument. The original version encompasses 17 items and evaluates the severity of depression during the last week [50]. Nine items are scored on a 5-point scale ranging from 0 to 4, and eight items are scored on a 3-point scale ranging from 0 to 2. A score equal to or below 7 is indicative of remission or absence of depression and the total score can range from 0 to a maximum of 52 points [51]. There are different recommendations regarding classification of depression severity using HAM-D scores and no consensus has been reached [49]. According to Zimmerman et al. (2013), a HAM-D score of 8–16 indicates symptoms of mild intensity while a score of 17–23 shows a moderate depression severity. A score ≥24 corresponds to a severe depressive episode [10].

The WHOQOL-BREF is a 26-item self-administered and cross-cultural scale used to assess quality of life and is an abbreviated version of the WHOQOL-100 scale. WHOQOL-BREF is comprised of four domains: physical health (7 items), psychological health (6 items), social relationships (3 items), and environmental health (8 items). Items are rated on a 5-point Likert scale that ranges from 1 (very dissatisfied) to 5 (very satisfied) and a higher score indicates a better subjective quality of life [14]. The raw domain scores were transformed to a 0 to 100 scale according to scoring guidelines [52]. WHOQOL-BREF is a generic scale but has been successfully used to measure quality of life in patients with depression and was reported as sensitive to improvement due antidepressant treatment [53,54]. The scale was validated on the Romanian population and has been reported to have good psychometric proprieties [55].

The VAS for pain is a simple and rapid measure to assess subjective pain intensity and has been used in a wide range of populations, including MDD patients with painful physical symptoms [56,57,58]. The scale used in this study was represented by a 10 cm length horizontal line anchored at both ends by two verbal descriptors of pain intensity, more precisely “no pain” and “pain as bad as it could be” [56]. Patients were asked to draw a perpendicular line on the VAS line where they consider that it best represents their overall pain intensity during the past week. The score ranges from 0 to 10 and it was calculated using a ruler measured in millimeters.

SDS is a simple, self-administered scale which encompass three items that assess work/school, social life, and family/home responsibilities impairment due to emotional symptoms [59,60,61]. SDS is a discretized analogue scale that uses visual, numeric, and descriptive anchors simultaneously to quantify the impairment in functionality. The numbers range from 0 (not at all) to 10 (extremely). The total score is calculated by summing the three domains scores and ranges from 0 to 30 [61]. In addition, the degree of productivity impairment by number of days lost in the last week and the number of days underproductive in the last week were assessed using SDS [60]. SDS was extensively used in psychopharmacological trials as a measure of impairment [62].

#### 2.2.3. Statistical Analysis

Statistical analysis was performed using IBM Statistical Package for Social Sciences (SPSS) version 26.0. software for Windows (IBM, Armonk, NY, USA). Continuous variables were expressed as mean and standard deviation (mean ± SD), while categorical variables were expressed as absolute (number) and relative (percentage) frequency. Comparison between two continuous variables was made using paired *t*-test. Linear regression was used in order to test the independent predictive ability of sociodemographic and clinical factors for the change in WHOQOL-BREF domains scores. Simple univariate regression was conducted in order to test the individual relationship between the studied parameters and the outcomes. Multivariate linear regression models were built using a stepwise forward method. Factors were kept in the model if probability of F to enter was ≤0.05 and removed if probability of F to remove was ≥0.1. The dependent variable was the difference in domain score of WHOQOL-BREF between the two measuring points (i.e., change in score). The change in score of each domain was considered as an individual dependent variable. Variables tested for independent predictive ability were the sociodemographic (age, gender, level of education, marital status, place of residence, professional status, income) and clinical data (age at first diagnosis of depressive disorder, illness duration, history of suicide attempts, family psychiatric history, number of psychiatric hospitalizations, treatment before study enrolment), and the change in scores of body mass index (BMI), VAS, HAM-D, SDS, days lost, and days unproductive. Furthermore, collinearity between the factors introduced in the models was tested using tolerance and variance inflation factors. Effect size was expressed as adjusted R square. A *p* value less than an alpha level of 0.05 was considered statistically significant.

## 3. Results

**Figure 1:**
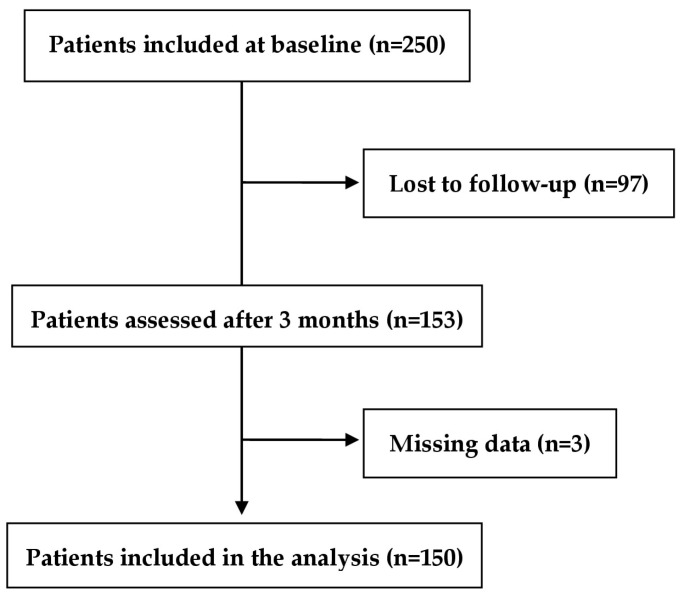
Flowchart of the study population.

For this study, 150 patients were included in the final analysis (Figure 1). The mean (±SD) age of the patients was 50.77 ± 10.17 years and 65.3% of the patients had between 9 to 12 years of formal education. No illiterate patients were included in the analysis. The mean (±SD) for the illness duration was 8.65 ± 9.35 years. Other sociodemographic and clinical characteristics of the patients are presented in Table 1.

The patients had a mean ± SD at T0 for the HAM-D of 19.73 ± 5.64 and at T1 of 11.47 ± 6.05. The mean ± SD of WHOQOL-BREF physical health, psychological, social relationships, and environmental domains at baseline was 43.94 ± 18.56, 49.27 ± 21.21, 52.64 ± 23.01, and 58.95 ± 16.67, respectively. At 3-month follow-up, the mean ± SD of WHOQOL-BREF physical health, psychological, social relationships, and environmental domains was 59.33 ± 19.56, 60.86 ± 20.86, 58.11 ± 20.89, and 64.65 ± 15.14, respectively. Other results from the psychometric instruments are presented in Table 2.

The changes in HAM-D and SDS scores were both negative statistically significant predictors for the change in physical health and psychological domains scores of WHOQOL-BREF (R^2^ = 0.451, *p* < 0.001; R^2^ = 0.325, *p* < 0.001, respectively; and R^2^ = 0.384, *p* < 0.001; R^2^ = 0.320, *p* < 0.001, respectively). The number of psychiatric hospitalizations was a negative predictor of physical health and psychological domains (R^2^ = 0.038, *p* < 0.01 and R^2^ = 0.027, *p* < 0.05, respectively). Other statistically significant predictors of the changes in scores of WHOQOL-BREF domains are presented in Table 3.

For each WHOQOL-BREF domain, we found a distinct model that predicts the change in score. The model, including HAM-D change, SDS change score, and VAS for pain change score, explained 54.7% (adjusted R^2^) of the variance of change in physical QoL score. The change in HAM-D score, change in SDS score, and marital status explained 41.2% (adjusted R^2^) of the variance of the change in psychological QoL score. The change in social relationships domain score was predicted by the model including the change in HAM-D score, change in SDS score, and history of suicide attempts. This model explained 17.7% (adjusted R^2^) of the variance of the change in social relationships QoL score. The change in score of environmental QoL was associated with reductions in depressive symptomatology and days lost and a lower level of education; this model explained 14.2% (adjusted R^2^) of the variance of the change in the environmental QoL score from baseline to 3-month follow-up (Table 4). See Supplementary Materials for the full multiple linear regression model of each change in WHOQOL-BREF domain score (Tables S1–S5).

## 4. Discussion

In the current study, it was demonstrated that decreases in the severity of depressive symptoms and of disability are associated with improvements in all facets of QoL. Moreover, a higher degree of reduction in days lost was associated with better improvements in all aspects of QoL as well. The results of this research showed that the greater the pain intensity alleviation, the greater the improvement in the physical aspect of QoL. In addition, an increased number of psychiatric hospitalizations predicted lower psychological and physical QoL changes but having a partner predicted better psychological and environmental QoL changes. Moreover, changes in HAM-D score, SDS score, and VAS overall pain score together explained 54.7% of the variance in physical health QoL changes from baseline to 3-month follow-up, while changes in HAM-D score and SDS score, and having a marital partner together captured 41.2% of the same variance. The variance in social relationships QoL changes was 17.7%, explained by the changes in HAM-D and SDS scores and by a positive history of suicide attempt. As much as 14.2% of the variance in environmental QoL changes are accounted for by the changes in HAM-D score and days lost, and a lower education level.

At baseline, the results regarding WHOQOL-BREF domain scores of our sample were similar to those of other studies conducted on MDD patients [30,53,63]. Moreover, compared to the international general population norms currently available, patients included in this research had noticeably lower scores [55]. This finding outlines once more that depressed patients have an impaired QoL.

Until now, several studies with a cross-sectional design have shown that severity of depressive symptomatology was a key determinant of QoL in different domains of MDD patients [7,17,63,64,65,66]. However, little research has evaluated the role of depressive severity improvement in predicting the change in different aspects of patient’s QoL. Cohen et al. (2013) and Pyne et al. (2003) found that changes in depressive symptoms during the acute treatment phase were associated with changes in patient’s perceptions of overall QoL (measured with Quality of Life Enjoyment and Satisfaction Questionnaire—Short Form and Quality of Well-Being self-administered version, respectively) [18,67], but changes in depressive symptomatology captured only 50% of the variance in QoL changes [18]. Morton et al. (2021) showed that symptoms reduction significantly predicted improvements over an eight-week period in psychological and physical domains of the WHOQOL-BREF scale, which is in agreement with the results of the present study [30]. As Morton et al. (2021) already argued, this is to be expected since some symptoms of depression (low mood and energy) are closely related to the psychological (negative feelings, self-esteem, thinking, learning, memory, and concentration) and physical health (energy and fatigue, sleep, and rest) QoL domains [30]. Our research also reported that decrease in depressive symptoms is a predictor of social relationships and environment domains. These results are to some extent in line with the earlier literature that found that greater depressive symptoms predict lower levels of QoL not only in the psychological and physical aspects but in the social relationships aspect as well during a flexible follow-up period of two to three years [68]. Moreover, the current study revealed that the change in HAM-D scores explained only a proportion of the variance in the change in WHOQOL-BREF scores; other variables such as the degree of disability, intensity of overall pain, or being in a marital partnership improved the predictive models for the change in time of the QoL aspects. Since QoL and the severity of depressive symptoms show a dynamic change over time, especially after treatment intervention, we argue that exploring the relationship between changes in scores is more important in order to identify which aspects can be improved during provision of care with the goal to normalize QoL. These results call for more efficient and multi-targeted interventions beyond symptoms remission in order to improve QoL as the ultimate outcome in MDD patients.

Increasing evidence suggests that large proportions of patients with MDD report painful symptoms [69,70,71,72]. Moreover, depressed patients with painful symptoms were observed to have a higher depression severity and worse QoL than those without these symptoms, and there seems to be a correlation between changes in depression scores and changes in pain intensity [73,74,75]. The results of our investigation showed that small decreases in overall pain intensity predicted poorer improvements in the physical health aspect of QoL. Moreover, changes in pain intensity was included in the final predictive model of physical health QoL, along with changes in SDS and HAM-D scores. These results are in line with those reported by an earlier study investigating pain (using VAS) and QoL (using Short Form Health Survey 36-item—SF-36) during a 3-month period. Chung et al. (2012) showed that the degree of pain reduction positively predicted the improvement in the physical component score of QoL [37]. In contrast, findings from the FINDER Study showed that even though patients with pain (VAS score > 30 mm) had lower QoL scores (SF-36) at all three time points (i.e., baseline, 3 and 6 months) than patients with mild or no pain, the pain variable was not a significant predictor of SF-36 change score from baseline to 6 months [76]. Among the plausible explanations for the heterogeneity of results are use of different QoL measurement instruments (which inherently offer particular insights of a broad and complex aspect) and study designs and the different follow-up periods. Moreover, unlike depressive symptoms, which are much better and objectively conceptualized, QoL is still a controversial and broad construct, and proper means of measuring it have not been agreed upon and are still debatable [54]. Even though the role of pain in determining the QoL of patients with MDD is supported by increased evidence, the role of pain reduction following treatment in predicting the change in QoL remains still unclear, and further research is needed to fill these knowledge gaps.

In addition, pain intensity reporting is suggested to be affected in patients suffering from chronic pain. Previous research has revealed that chronic pain patients have an impaired number sense in comparison with controls or acute pain patients, which is an important aspect to be considered when using visual scales to measure pain intensity [77,78,79]. Moreover, Spindler et al. (2018) proposed that impairments in working memory, which can be seen in depressed patients as well [80], might influence number sense [77]. However, VAS showed overall good stability and ability to detect changes in pain intensity over time in chronic pain patients [81]. In addition, although patients suffering from depression have an increased threshold of experimentally induced pain, they have a distinct processing profile characterized by a negative bias in the initial evaluation of pain intensity. This observation led to the possible conclusion that altered pain processing mediates the relationship between depression and pain complaints [82].

This study provides evidence that some sociodemographic characteristics of MDD patients are associated with the changes in QoL. A smaller number of psychiatric hospitalizations was a predictor of better physical health and psychological changes in QoL, but this variable was not included in the predictive models. According to Sørensen et al. (1996), psychiatric hospitalization is an effect variable that could serve as an indicator of QoL [16]. Therefore, the association reported in this study is to be inferred. Hospitalization represents an important source of patient burden that consequently contributes to an impaired QoL, and severity of symptoms is a well-known reason for MDD inpatient admission [83].

We also found that younger age was a predictor of better change in psychological QoL. Some studies showed that older age was associated with worse changes in the physical aspect of QoL [30,76] while Chung et al. (2012) reported that age was not a significant predictor of changes in any components (i.e., physical and mental) of the SF-36 instrument [37]. In our study, having a marital partner and a lower level of formal education predicted better changes in the psychological and environmental aspects of QoL, respectively, being in the predictive models of these domains. Conversely, being married was correlated with smaller positive changes in QoL over a 6-month period [76], and a higher education with better QoL [66]. Yang et al. (2021) found similar results with ours, namely, patients with lower education had greater improvements in QoL between baseline and 12-week follow-up [84]. While some studies found that educational level was not significantly associated with any of the WHOQOL-BREF domains or that less educated MDD patients had lower QoL, other studies did not include the educational level among collected variables [7,17,63]. Potential explanations for our data reside in the sampling selection and classification of individuals’ educational attainment or marital status. All of these aspects contribute to the existing body of conflicting results regarding the role of several sociodemographic factors in determining QoL and call for further in-depth research.

An interesting result of this study is the association between a positive history of suicide attempts and a better change in QoL in the social relationships domain. Similar results were obtained by Yang et al. (2021), which reported that patients with severe suicidal ideation had a substantial improvement of QoL over a 3-month period [84]. Findings from the STAR*D trial have shown that major depressive patients with a history of suicide attempts had a more severe course of illness (i.e., number of episodes, age at onset, severity of depression) [85]. Moreover, according to Yang et al. (2021), patients with more severe symptoms have a greater improvement of depression after acute phase treatment [84]. Moreover, a greater symptom reduction is associated with a larger improvement in QoL, as shown by the current study. Therefore, since history of suicide attempts echoes severe depression, it will determine an increased change in QoL during the 3-month treatment phase.

The present study suggests that reduction in disability was a significant predictor across all investigated domains of QoL. Moreover, the change in SDS score was included in the predictive models for social relationships, physical health, and psychological aspects of QOL, contributing most significantly to the variance of the latter two. There is scarce evidence of the role of functionality in determining changes in the QoL of depressive patients. One potential explanation is related to the similarities between the two concepts despite their distinctive characteristics and purposes (i.e., one measures the degree of disability in properly accomplishing various roles due to a disease, while the other evaluates subjective satisfaction with one’s different life aspects) [45]. There is scarce evidence regarding the impact of changes in functionality on changes in QoL. Most of current data come from cross-sectional studies that reported an association between disability and overall QoL. Results reported by Morton et al. (2021) were similar to ours [30].

An interesting result of this research is that BMI is a positive predictor of changes in psychological and physical health QoL, meaning that an increase in BMI is associated with improvements in QoL. MDD can determine a decrease in appetite that can even result in unintentional weight loss. Such complaints are included in the core of symptoms of MDD and are among the most frequent somatic symptoms reported by depressed patients [86]. Therefore, an increase in BMI during antidepressant pharmacotherapy indicates an improvement of depressive symptoms which is strongly associated with positive changes in QoL. Moreover, we hypothesize that through improving their appetite, and therefore having an increase in BMI, patients might have a more positive view of their physical health.

This study has several limitations that should be acknowledged. Firstly, the sample size is relatively small; therefore, future larger representative studies could shed more light on the path of change in QoL over time. Moreover, this research included patients with different courses of illness; therefore, these results cannot mirror a particular subtype of patients suffering from MDD. Additionally, even though we aimed at assessing as many variables as possible that had been explored separately up to this point, we are aware that we did not assess all factors that might be involved in shaping QoL. Regarding pain, we evaluated the intensity but not the presence of painful symptoms, which could be considered a further limitation. Future studies should also include a supplementary subjective assessment of depressive symptoms since QoL is a subjective measure as well. Finally, another limitation of the current study is the follow-up period that encompassed only the acute phase of the treatment. We believe that follow-ups during longer terms could provide more insights into the changes in the QoL of patients with MDD, thus paving the way towards better personalized care.

## 5. Conclusions

Notwithstanding the study limitations, we conclude that the changes in the QoL of MDD subjects is directly affected by several sociodemographic factors and by the changes in pain intensity, functionality, and severity of symptoms as well. The predictive models built by this research show that even though for some aspects of the QoL (i.e., physical health and psychological health), symptoms’ intensity contributed significantly to the variance of the QoL change, there are other factors that might interfere with changes in QoL during the short-term treatment phase. Further research focusing on these factors is needed in order to fill in the gaps in current research. To the best of our knowledge, this is among the few studies to have explored the combined impact of pain, functionality, and depression severity on QoL outcomes. Moreover, this research emphasizes the importance of comprehensive assessment of patients suffering from MDD beyond symptomatology and of including measures of QoL in everyday practice with the aim of offering tailored interventions for individual needs.

## Figures and Tables

**Table 1:** Sociodemographic and clinical characteristics at baseline of patients (N = 150) included in the analysis.

Patient Characteristics	
Age (years), mean ± SD	50.77 ± 10.17
Gender, N (%)	
Males	45 (30.0%)
Females	105 (70.0%)
Level of education, N (%)	
≤8 years	30 (20.0%)
9–12 years	98 (65.3%)
>12 years	22 (14.7%)
Marital Status, N (%)	
With Partner	47 (31.3%)
Single	103 (68.7%)
Place of Residence, N (%)	
Rural	52 (34.7%)
Urban	98 (65.3%)
Professional Status, N (%)	
Employed	52 (34.7%)
Retired or Unemployed	98 (65.3%)
Income, N (%)	
Low	35 (23.3%)
Medium	92 (61.3%)
High	23 (15.3%)
Age at first diagnosis of depressive disorder, mean ± SD	42.21 ± 12.37
Illness Duration (years), mean ± SD	8.65 ± 9.35
History of Suicide Attempts, N (%)	
No	114 (76.0%)
Yes	36 (24.0%)
Family Psychiatric History, N (%)	
Yes	50 (33.3%)
No	100 (66.7%)
Number of psychiatric hospitalizations, mean ± SD	5.84 ± 11.53
Treatment before study enrolment, N (%)	
Yes	122 (81.3%)
No	28 (18.7%)
Level of depressive symptoms severity, N (%)	
Mild	42 (28%)
Moderate	63 (42%)
Severe	45 (30%)

N, number of patients; SD, standard deviation.

**Table 2:** Psychometric scales scores, days lost, days unproductive and BMI at T0 and T1.

Measure	T0	T1	p *	Change in Score (Difference between T1 and T0)
HAM-D	19.73 ± 5.64	11.47 ± 6.05	0.000	−8.25 ± 5.93
WHOQOL-BREF domain				
Physical health	43.94 ± 18.56	59.33 ± 19.56	0.000	15.39 ± 17.00
Psychological	49.27 ± 21.21	60.86 ± 20.86	0.000	11.58 ± 17.79
Social relationships	52.64 ± 23.01	58.11 ± 20.89	0.000	5.47 ± 16.86
Environmental	58.95 ± 16.67	64.65 ± 15.14	0.000	5.71 ± 8.73
SDS	19.45 ± 9.14	8.43 ± 9.30	0.000	−11.02 ± 9.21
VAS for pain	4.74 ± 3.45	4.12 ± 3.49	0.020	−0.63 ± 3.63
Days Lost	2.46 ± 2.79	0.49 ± 1.31	0.000	−1.97 ± 2.66
Days Unproductive	2.38 ± 2.53	1.97 ± 2.64	0.121	−0.41 ±3.10
BMI	27.16 ± 5.35	27.87 ± 5.42	0.000	0.70 ± 1.73

Data are presented as mean ± SD, unless otherwise indicated; HAM-D, Hamilton Depression Rating Scale; SDS, Sheehan Disability Scale; VAS, visual analogue scale; WHOQOL-BREF, World Health Organization Quality of Life scale—abbreviated version; *, paired *t*-test.

**Table 3:** Univariate linear regressions results showing the statistically significant predictors of the change in WHOQOL-BREF domains scores (i.e., from baseline to 3-month follow-up).

Predictor	B	t	*p*	Adjusted R^2^
**Physical health**				
Number of psychiatric hospitalizations	−0.313	−2.630	0.009	0.038
Change in BMI score	2.109	2.679	0.008	0.040
Change in VAS for pain score	−1.731	−4.832	0.000	0.130
Change in HAM-D score	−1.934	−11.119	0.000	0.451
Change in SDS score	−1.149	−9.681	0.000	0.384
Change in days lost score	−2.302	−4.707	0.000	0.124
Change in days unproductive score	−1.660	−3.868	0.000	0.086
**Psychological**				
Age	−0.351	−2.495	0.014	0.034
Marital status	6.861	2.219	0.028	0.026
Number of psychiatric hospitalizations	−0.285	−2.270	0.025	0.027
Change in BMI score	2.172	2.634	0.009	0.038
Change in HAM-D score	−1.722	−8.530	0.000	0.325
Change in SDS score	−1.099	−8.424	0.000	0.320
Change in days lost score	−2.014	−3.851	0.000	0.085
Change in days unproductive score	−1.735	−3.860	0.000	0.085
**Social relationships**				
History of suicide attempts	6.879	2.160	0.032	0.024
Change in HAM-D score	−1.049	−4.830	0.000	0.130
Change in SDS score	−0.673	−4.814	0.000	0.130
Change in days lost score	−1.255	−2.462	0.015	0.033
**Environment**				
Level of education	−2.489	−2.069	0.040	0.022
Marital status	3.495	2.306	0.023	0.028
Change in HAM-D score	−0.466	−4.059	0.000	0.094
Change in SDS score	−0.293	−3.952	0.000	0.089
Change in days lost score	−0.898	−3.466	0.001	0.069

HAM-D, Hamilton Depression Rating Scale; SDS, Sheehan Disability Scale; VAS, visual analogue scale; WHOQOL-BREF, World Health Organization Quality of Life scale—abbreviated version.

**Table 4:** Stepwise multiple linear regressions results showing the summary of each model that predicted the change scores of WHOQOL-BREF domains (i.e., from baseline to 3-month follow-up).

Model	R	R^2^	Adjusted R^2^	R^2^ Change	F Change	*p* F Change
**Physical health**						
(Constant), change in HAM-D score, change in SDS score, change in VAS score	0.746	0.556	0.547	0.038	12.398	0.001
**Psychological**						
(Constant), change in HAM-D score, change in SDS score, marital status	0.651	0.424	0.412	0.025	6.206	0.014
**Social relationships**						
(Constant), change in HAM-D score, change in SDS score, history of suicide attempts	0.440	0.194	0.177	0.026	4.638	0.033
**Environment**						
(Constant), change in HAM-D score, educational level, change in days lost score	0.399	0.160	0.142	0.027	4.598	0.034

HAM-D, Hamilton Depression Rating Scale; SDS, Sheehan Disability Scale; VAS, visual analogue scale; WHOQOL-BREF, World Health Organization Quality of Life scale—abbreviated version.

## Data Availability

The data presented in this study are available on reasonable request from the corresponding author. The data are not publicly available due to ethical and institutional reasons.

## Supplementary Materials

The following supporting information can be downloaded at: https://www.mdpi.com/article/10.3390/jcm12144628/s1, Tables S1–S5: Full multiple linear regressions model for each change in WHOQOL-BREF domain score.
