# Supplementary material for: Predictors of Changes in Quality of Life of Patients with Major Depressive Disorder—A Prospective Naturalistic 3-Month Follow-Up Study
Source: J Clin Med. 2023 Jul 12;12(14):4628. doi: 10.3390/jcm12144628 (PMC10380991; doi:10.3390/jcm12144628)
Supplement: Supplementary file 1 [file jcm-12-04628-s001.zip › jcm-2465280-supplementary.pdf]

Supplementary Material

Full multiple linear regression model for each WHOQOL-BREF domain
Table S1

	R	R^2	Adjusted R^2	R^2 change	F change	*p* F change
**Physical health**						
Step 1. Predictors: (Constant), HAM-D score evolution.	,674	,454	,450	,454	122,104	,000
Step 2. Predictors: (Constant), HAM-D score evolution, SDS score evolution.	,720	,518	,511	,064	19,394	,000
Step 3 – final model. Predictors: (Constant), HAM-D score evolution, SDS score evolution, VAS score evolution	,746	,556	,547	,038	12,398	,001
**Psychological**						
Step 1. Predictors: (Constant), HAM-D score evolution	,573	,328	,324	,328	71,775	,000
Step 2. Predictors: (Constant), HAM-D score evolution, SDS score evolution.	,632	,399	,391	,071	17,188	,000
Step 3 – final model. Predictors: (Constant), HAM-D score evolution, SDS score evolution, marital status	,651	,424	,412	,025	6,206	,014
**Social relationships**						
Step 1. Predictors: (Constant), HAM-D score evolution	,370	,137	,131	,137	23,377	,000
Step 2. Predictors: (Constant), HAM-D score evolution, SDS score evolution.	,410	,168	,157	,031	5,401	,022
Step 3 – final model. Predictors: (Constant), HAM-D score evolution, SDS score evolution, suicide attempts	,440	,194	,177	,026	4,638	,033
**Environment**						
Step 1. Predictors: (Constant), HAM-D score evolution	,316	,100	,094	,100	16,269	,000
Step 2. Predictors: (Constant), HAM-D score evolution, educational level	,365	,133	,121	,033	5,606	,019
Step 3 – final model. Predictors: (Constant), HAM-D score evolution, educational level, days lost evolution	,399	,160	,142	,027	4,598	,034

Table S2

Excluded Variables – Dependent variable = Physical Health domain change score					
Step		Beta In	t	Sig.	Partial Correlation
1	Gender	,106[b]	1,712	,089	,140
	Age	-,007[b]	-,111	,911	-,009
	Educational level	-,014[b]	-,232	,816	-,019
	Marital status	,070[b]	1,144	,255	,094
	Professional status	,030[b]	,494	,622	,041
	Income	-,043[b]	-,702	,484	-,058
	Place of residence	-,012[b]	-,189	,850	-,016
	Illness duration	-,085[b]	-1,395	,165	-,115
	Age at first diagnosis of depressive disorder	,071[b]	1,135	,258	,093
	Number of psychiatric hospitalizations	-,121[b]	-1,989	,049	-,162
	History of suicide attempts	,030[b]	,493	,623	,041
	Family psychiatric history	,021[b]	,342	,733	,028
	Treatment before study enrolment	,051[b]	,833	,406	,069
	BMI change score	,115[b]	1,885	,061	,154
	VAS change score	-,207[b]	-3,390	,001	-,270
	SDS change score	-,326[b]	-4,404	,000	-,342
	Days lost change score	-,146[b]	-2,287	,024	-,186
	Days unproductive change score	-,081[b]	-1,254	,212	-,103
2	Gender	,092[c]	1,584	,115	,130
	Age	,014[c]	,239	,812	,020
	Educational level	-,021[c]	-,365	,715	-,030
	Marital status	,104[c]	1,799	,074	,148
	Professional status	,029[c]	,497	,620	,041
	Income	-,066[c]	-1,146	,254	-,095
	Place of residence	-,009[c]	-,148	,883	-,012
	Illness duration	-,066[c]	-1,150	,252	-,095
	Age at first diagnosis of depressive disorder	,075[c]	1,278	,203	,106
	Number of psychiatric hospitalizations	-,115[c]	-1,999	,047	-,164
	History of suicide attempts	,047[c]	,816	,416	,068
	Family psychiatric history	-,027[c]	-,453	,651	-,038
	Treatment before study enrolment	,030[c]	,508	,612	,042
	BMI change score	,106[c]	1,833	,069	,150
	VAS change score	-,202[c]	-3,521	,001	-,281
	Days lost change score	-,068[c]	-1,063	,290	-,088
	Days unproductive change score	-,030[c]	-,476	,635	-,040
3	Gender	,093[d]	1,660	,099	,137
	Age	,017[d]	,301	,764	,025
	Educational level	,016[d]	,277	,782	,023
	Marital status	,108[d]	1,952	,053	,161
	Professional status	,032[d]	,568	,571	,047
	Income	-,070[d]	-1,265	,208	-,105
	Place of residence	-,011[d]	-,206	,837	-,017
	Illness duration	-,053[d]	-,948	,345	-,079
	Age at first diagnosis of depressive disorder	,070[d]	1,238	,218	,103
	Number of psychiatric hospitalizations	-,100[d]	-1,796	,075	-,148
	Number of suicide attempts	,054[d]	,968	,335	,080
	Family psychiatric history	-,023[d]	-,412	,681	-,034
	Treatment before study enrolment	,049[d]	,863	,390	,072
	BMI change score	,109[d]	1,972	,051	,162
	Days lost change score	-,068[d]	-1,098	,274	-,091
	Days unproductive change score	-,026[d]	-,432	,667	-,036

Table S3

Excluded Variables – Dependent variable = Psychological domain change score					
Step		Beta In	t	p	Partial Correlation
1	Gender	-,011[b]	-,156	,876	-,013
	Age	-,083[b]	-1,208	,229	-,099
	Educational level	-,084[b]	-1,242	,216	-,102
	Marital status	,123[b]	1,819	,071	,149
	Professional status	,050[b]	,734	,464	,061
	Income	-,075[b]	-1,105	,271	-,091
	Place of residence	,046[b]	,677	,499	,056
	Illness duration	-,110[b]	-1,627	,106	-,133
	Age at first diagnosis of depressive disorder	,028[b]	,398	,691	,033
	Number of psychiatric hospitalizations	-,107[b]	-1,574	,118	-,129
	History of suicide attempts	,052[b]	,761	,448	,063
	Family psychiatric history	,058[b]	,859	,392	,071
	Treatment before study enrolment	,070[b]	1,019	,310	,084
	BMI change score	,127[b]	1,877	,063	,153
	VAS change score	,033[b]	,467	,641	,039
	SDS change score	-,343[b]	-4,146	,000	-,325
	Days lost change score	-,118[b]	-1,651	,101	-,135
	Days unproductive change score	-,121[b]	-1,682	,095	-,138
2	Gender	-,025[c]	-,381	,704	-,032
	Age	-,062[c]	-,937	,350	-,078
	Educational level	-,091[c]	-1,423	,157	-,117
	Marital status	,159[c]	2,491	,014	,203
	Professional status	,048[c]	,748	,455	,062
	Income	-,099[c]	-1,548	,124	-,127
	Place of residence	,049[c]	,764	,446	,063
	Illness duration	-,090[c]	-1,403	,163	-,116
	Age at first diagnosis of depressive disorder	,032[c]	,488	,626	,040
	Number of psychiatric hospitalizations	-,100[c]	-1,554	,122	-,128
	History of suicide attempts	,070[c]	1,079	,282	,089
	Family psychiatric history	,010[c]	,146	,884	,012
	Treatment before study enrolment	,047[c]	,720	,472	,060
	BMI change score	,117[c]	1,821	,071	,150
	VAS change score	,038[c]	,571	,569	,047
	Days lost change score	-,032[c]	-,449	,654	-,037
	Days unproductive change score	-,068[c]	-,972	,333	-,080
3	Gender	-,030[d]	-,467	,641	-,039
	Age	-,042[d]	-,649	,517	-,054
	Educational level	-,078[d]	-1,232	,220	-,102
	Professional status	,035[d]	,545	,586	,045
	Income	-,087[d]	-1,373	,172	-,114
	Place of residence	,044[d]	,694	,489	,058
	Illness duration	-,090[d]	-1,428	,156	-,118
	Age at first diagnosis of depressive disorder	,051[d]	,790	,431	,066
	Number of psychiatric hospitalizations	-,089[d]	-1,393	,166	-,115
	Number of suicide attempts	,053[d]	,829	,409	,069
	Family psychiatric history	,007[d]	,106	,916	,009
	Treatment before study enrolment	,050[d]	,780	,437	,065
	BMI change score	,115[d]	1,813	,072	,149
	VAS change score	,034[d]	,525	,600	,044
	Days lost change score	-,013[d]	-,186	,853	-,016
	Days unproductive change score	-,069[d]	-1,013	,313	-,084

Table S4

Excluded Variables – Dependent variable = Social relationship domain change score					
Model		Beta In	t	Sig.	Partial Correlation
1	Gender	,073[b]	,940	,349	,078
	Age	-,070[b]	-,897	,371	-,074
	Educational level	-,145[b]	-1,909	,058	-,156
	Marital status	,095[b]	1,237	,218	,102
	Professional status	,059[b]	,764	,446	,063
	Income	-,058[b]	-,755	,451	-,062
	Place of residence	,005[b]	,064	,949	,005
	Illness duration	-,016[b]	-,203	,840	-,017
	Age at first diagnosis of depressive disorder	-,057[b]	-,726	,469	-,060
	Number of psychiatric hospitalizations	,103[b]	1,333	,185	,110
	History of suicide attempts	,149[b]	1,959	,052	,160
	Family psychiatric history	,007[b]	,094	,925	,008
	Treatment before study enrolment	,085[b]	1,097	,275	,090
	BMI change score	,101[b]	1,307	,193	,108
	VAS change score	,021[b]	,258	,797	,021
	SDS change score	-,226[b]	-2,324	,022	-,189
	Days lost change score	-,083[b]	-1,017	,311	-,084
	Days unproductive change score	,023[b]	,275	,784	,023
2	Gender	,064[c]	,833	,406	,069
	Age	-,056[c]	-,723	,471	-,060
	Educational level	-,150[c]	-2,004	,047	-,164
	Marital status	,119[c]	1,569	,119	,129
	Professional status	,058[c]	,761	,448	,063
	Income	-,074[c]	-,979	,329	-,081
	Place of residence	,007[c]	,093	,926	,008
	Illness duration	-,002[c]	-,032	,975	-,003
	Age at first diagnosis of depressive disorder	-,054[c]	-,698	,486	-,058
	Number of psychiatric hospitalizations	,107[c]	1,413	,160	,117
	History of suicide attempts	,161[c]	2,154	,033	,176
	Family psychiatric history	-,026[c]	-,336	,737	-,028
	Treatment before study enrolment	,070[c]	,916	,361	,076
	BMI change score	,095[c]	1,239	,217	,102
	VAS change score	,024[c]	,306	,760	,025
	Days lost change score	-,027[c]	-,317	,751	-,026
	Days unproductive change score	,061[c]	,746	,457	,062
3	Gender	,051[d]	,667	,506	,055
	Age	-,026[d]	-,339	,735	-,028
	Educational level	-,124[d]	-1,636	,104	-,135
	Marital status	,103[d]	1,357	,177	,112
	Professional status	,033[d]	,428	,670	,036
	Income	-,064[d]	-,846	,399	-,070
	Place of residence	,011[d]	,140	,889	,012
	Illness duration	-,038[d]	-,497	,620	-,041
	Age at first diagnosis of depressive disorder	-,006[d]	-,080	,936	-,007
	Number of psychiatric hospitalizations	,049[d]	,587	,558	,049
	Family psychiatric history	-,030[d]	-,391	,696	-,033
	Treatment before study enrolment	,063[d]	,825	,411	,069
	BMI change score	,098[d]	1,306	,193	,108
	VAS change score	,018[d]	,237	,813	,020
	Days lost change score	,008[d]	,090	,928	,008
	Days unproductive change score	,028[d]	,334	,739	,028

Table S5

Excluded Variables – Dependent variable = Environment domain change score					
Model		Beta In	t	Sig.	Partial Correlation
1	Gender	,062[b]	,776	,439	,064
	Age	-,064[b]	-,795	,428	-,066
	Educational level	-,183[b]	-2,368	,019	-,192
	Marital status	,156[b]	2,004	,047	,164
	Professional status	,076[b]	,966	,336	,080
	Income	-,120[b]	-1,533	,127	-,126
	Place of residence	,000[b]	,001	1,000	,000
	Illness duration	-,040[b]	-,510	,611	-,042
	Age at first diagnosis of depressive disorder	-,029[b]	-,363	,717	-,030
	Number of psychiatric hospitalizations	,024[b]	,308	,758	,026
	History of suicide attempts	,085[b]	1,081	,282	,089
	Family psychiatric history	-,090[b]	-1,148	,253	-,095
	Treatment before study enrolment	-,058[b]	-,734	,464	-,061
	BMI change score	,047[b]	,591	,555	,049
	VAS change score	,049[b]	,599	,550	,050
	SDS change score	-,181[b]	-1,807	,073	-,148
	Days lost change score	-,187[b]	-2,281	,024	-,186
	Days unproductive change score	,057[b]	,678	,499	,056
2	Gender	,057[c]	,724	,470	,060
	Age	-,054[c]	-,679	,498	-,056
	Marital status	,141[c]	1,823	,070	,150
	Professional status	,003[c]	,038	,969	,003
	Income	-,062[c]	-,743	,459	-,062
	Place of residence	,037[c]	,466	,642	,039
	Illness duration	-,068[c]	-,867	,387	-,072
	Age at first diagnosis of depressive disorder	-,004[c]	-,048	,962	-,004
	Number of psychiatric hospitalizations	-,015[c]	-,186	,853	-,015
	History of suicide attempts	,051[c]	,652	,515	,054
	Family psychiatric history	-,121[c]	-1,549	,123	-,128
	Treatment before study enrolment	-,038[c]	-,487	,627	-,040
	BMI change score	,063[c]	,800	,425	,066
	VAS change score	,087[c]	1,066	,288	,088
	SDS change score	-,187[c]	-1,903	,059	-,156
	Days lost change score	-,174[c]	-2,144	,034	-,175
	Days unproductive change score	,028[c]	,332	,740	,028
3	Gender	,052[d]	,665	,507	,055
	Age	-,038[d]	-,480	,632	-,040
	Marital status	,132[d]	1,723	,087	,142
	Professional status	-,002[d]	-,028	,978	-,002
	Income	-,072[d]	-,872	,384	-,073
	Place of residence	,039[d]	,493	,623	,041
	Illness duration	-,069[d]	-,889	,375	-,074
	Age at first diagnosis of depressive disorder	,005[d]	,065	,948	,005
	Number of psychiatric hospitalizations	-,025[d]	-,319	,750	-,027
	Number of suicide attempts	,027[d]	,344	,731	,029
	Family psychiatric history	-,121[d]	-1,581	,116	-,131
	Treatment before study enrolment	-,045[d]	-,575	,566	-,048
	BMI change score	,069[d]	,893	,373	,074
	VAS change score	,086[d]	1,066	,288	,088
	SDS change score	-,134[d]	-1,299	,196	-,108
	Days unproductive change score	-,094[d]	-,969	,334	-,080
